# Comparison of Simple Eudragit Microparticles Loaded with Prednisolone and Eudragit-Coated Chitosan-Succinyl-Prednisolone Conjugate Microparticles: Part II. *In Vivo* Evaluation of Efficacy, Toxicity, and Biodisposition Characteristics

**DOI:** 10.3390/ijms161125949

**Published:** 2015-11-02

**Authors:** Hiraku Onishi, Hisashi Kikuchi

**Affiliations:** Department of Drug Delivery Research, Hoshi University, 2-4-41 Ebara, Shinagawa-ku, Tokyo 142-8501, Japan; onsh-hrk@ezweb.ne.jp

**Keywords:** simple Eudragit S100 microparticle, Eudragit S100-coated chitosan-succinyl-prednisolone conjugate microparticle, efficacy, toxicity, pharmacokinetic characteristics, 2,4,6-trinitrobenzenesulfonic acid-induced

## Abstract

We previously prepared and evaluated simple Eudragit S100 microparticles loaded with prednisolone (ES-MP) and Eudragit S100-coated chitosan-succinyl-prednisolone conjugate microparticles (Ch-MP/ES) *in vitro*. In this work, the effectiveness, toxic side effects (5 mg prednisolone (PD) eq/kg × 3 d, 10 mg PD eq/kg × 3 d), and pharmacokinetic characteristics (5 mg PD eq/kg) were examined using rats with colitis induced through 2,4,6-trinitrobenzenesulfonic acid. ES-MP did not change the efficacy or toxic side effects of PD, and this was attributed to incomplete delivery to the target site and prolonged systemic drug absorption by ES-MP. On the other hand, Ch-MP/ES promoted the efficacy of PD and ameliorated its toxic side effects due to better delivery to the target site, very slow drug release and the strong suppression of drug absorption. Only Ch-MP/ES, which markedly changed drug release characteristics, improved the *in vivo* features of PD.

## 1. Introduction

Ulcerative colitis (UC) is a chronic and refractory disease, and the number of the patients is large in various developed countries [1,2]. It is difficult to cure UC completely; therefore, UC has become a serious health issue. Although antibodies against pro-inflammatory cytokines have recently been developed as novel and highly potent agents [3,4], conventional drugs including 5-aminosasalicylic acid and glucocorticoids are still frequently used to patients with UC [5,6,7]. Prednisolone (PD) is an important drug that is used to treat moderate and severe UC. Conventional anti-inflammatory drugs, including PD, often cause toxic side effects because of their long-term repeated use [8,9]. Recent reports implicated the long-term administration of PD in the development of osteoporosis, diabetes, and increased susceptibility to infection [10,11]. Since these toxic side effects are attributed to systemic absorption and lower selectivity to the disease area, the drug targeting to the diseased site has been suggested to elevate its efficacy and reduce the severity of associated toxic side effects. Many delivery systems to the UC diseased area have been developed including those using pH-sensitive polymers [12], prolonged release through matrix systems [13,14], pro-drugs [15], and timed-release systems [16].

We previously developed two types of PD delivery systems to the lower intestine [17]: Ones is simple PD-loaded microparticles produced with Eudragit S100 (ES), named ES-MP, and the other is ES-coated microparticles prepared with chitosan-succinyl-prednisolone conjugate, called Ch-MP/ES). Their release profiles were investigated *in vitro* [17]. The release of PD from ES-MP was suppressed at gastric pH and gradual over 24 h at small intestinal pH; however, its release from Ch-MP/ES was markedly slower than that from ES-MP. In the present study, the efficacy, toxicity and biodisposition characteristics of these microparticles were compared *in vivo* in order to determine their advantages.

## 2. Results and Discussion

### 2.1. Particle Characteristics of ES-MP, Ch-MP, and Ch-MP/ES

**Figure 1:**
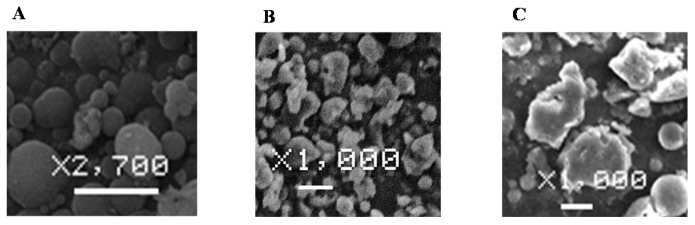
Scanning electron micrographs of (**A**) ES-MP; (**B**) Ch-MP and (**C**) Ch-MP/ES. The length of the white bar = 5 µm.

In this study, simple ES microparticles (ES-MP), chitosan-succinyl-prednisolone conjugate microparticles (Ch-MP) and ES-coated Ch-MP (Ch-MP/ES) were prepared. Their particle size and shape were observed by scanning electron microscopy. The obtained micrographs are shown in Figure 1.

ES-MP and had an almost spherical shape with a smooth surface. Ch-MP and Ch-MP/ES had a rough surface and were in a subglobular or ellipsoidal form. Ch-MP/ES were several-fold larger than Ch-MP because multiple Ch-MP could be contained in one Ch-MP/Ch, as reported previously [17]. Drug contents were obtained as shown in Table 1.

**Table 1:** Particle characteristics of ES-MP, Ch-MP and Ch-MP/ES.

Microparticles	PD Content (%, *w*/*w*)	Particle Size (µm)
ES-MP	3.73 ± 0.74	1.2 ± 0.3
Ch-MP	5.44 ± 0.17	2.8 ± 2.2
Ch-MP/ES	2.27 ± 0.27	18.7 ± 12.3

### 2.2. Evaluation of Efficacy and Toxicity

**Figure 2:**
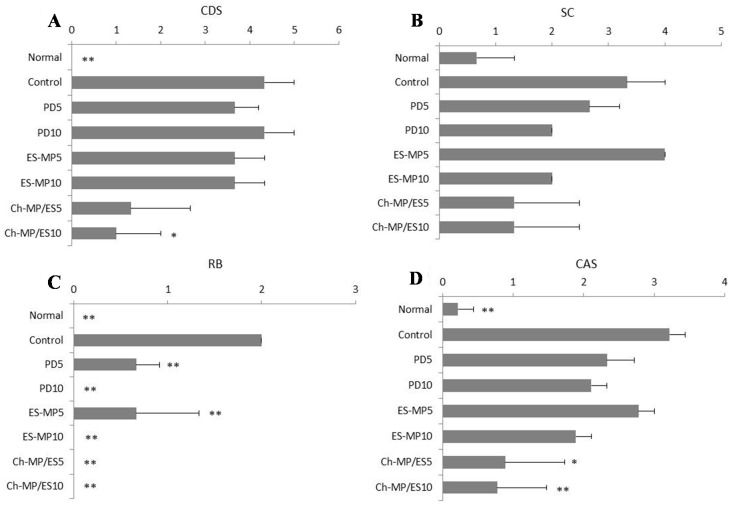
CDS, SC, RB and CAS after oral administration of each formulation in rats with TNBS-induced colitis. Efficay was evaluated from (**A**) CDS; (**B**) SC; (**C**) RB and (**D**) CAS. Normal (healthy), Control (non-treated), PD5 (5 mg PD/kg × 3 times (every 24 h)), PD10 (5 mg PD/kg × 6 times (every 12 h)), ES-MP5 (5 mg PD eq/kg × 3 times (every 24 h)), ES-MP10 (5 mg PD eq/kg × 6 times (every 12 h)), Ch-MP/ES5 (5 mg PD eq/kg × 3 times (every 24 h)), and Ch-MP/ES10 (5 mg PD eq/kg × 6 times (every 12 h)) were examined. The results are described as the mean ± S.E. (*n* = 3). * *p* < 0.05, ** *p* < 0.01 *vs.* Control. CAS = (CDS + SC + RB)/3.

**Figure 3:**
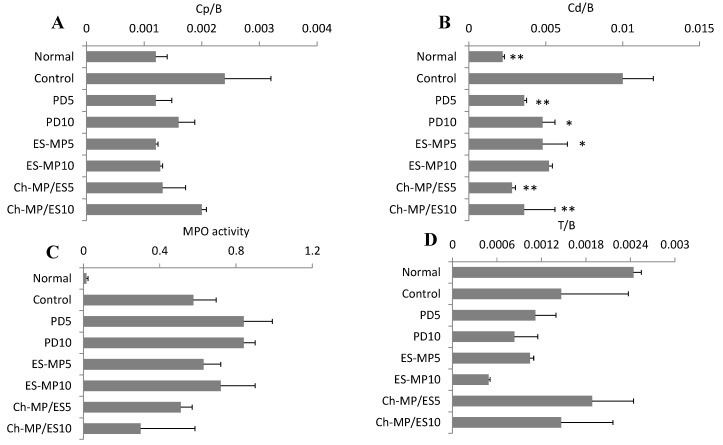
Cp/B, Cd/B, MPO activity and T/B after oral administration of each formulation in rats with TNBS-induced colitis. Efficacy was evaluated from (**A**) Cp/B values; (**B**) Cd/B values and (**C**) MPO activity; Toxic side effect was investigated from (**D**) T/B values. Normal (healthy), Control (non-treated), PD5 (5 mg PD/kg × 3 times (every 24 h)), PD10 (5 mg PD/kg × 6 times (every 12 h)), ES-MP5 (5 mg PD eq/kg × 3 times (every 24 h)), ES-MP10 (5 mg PD eq/kg × 6 times (every 12 h)), Ch-MP/ES5 (5 mg PD eq/kg × 3 times (every 24 h)), and Ch-MP/ES10 (5 mg PD eq/kg × 6 times (every 12 h)) were investigated. The results are described as the mean ± S.E. (*n* = 3). * *p* < 0.05, ** *p* < 0.01 *vs.* Control.

*In vitro* release studies were reported previously for ES-MP and Ch-MP/ES [17]; the release of PD from both microparticles was smell at gastric pH (pH 1.2) and gentle at small intestinal pH (pH 6.8). The release of PD from Ch-MP/ES was markedly slower due to its release mechanism of chemical hydrolysis of the ester. The purpose of this study is to evaluate the efficacy and toxicity of PD in ES-MP and Ch-MP/ES *in vivo*, in which rats with colitis produced using 2,4,6-trinitrobenzenesulfonic acid (TNBS) were used (Figure 2 and Figure 3).

Efficacy was first evaluated from the visual observation of the severity of colonic damage and inflammation. That is, the distal colon was washed briefly using saline to remove its contents, and the resultant distal colon was cut open and the severity of colonic damage and inflammation was evaluated by referring to the methods used by Lamprecht *et al.* [13] or Tozaki *et al.* [18]. The colonic damage of the specimen was scored as colonic damage score (CDS) from 0–5 as follows; score is 0 for no damage, 1 for localized hyperemia but no ulcer, 2 for linear ulcers with no significant inflammation. 3 for linear ulcer with inflammation at one site, 4 for two or more sites of ulceration and/or inflammation, and 5 for two or more major site of inflammation and ulceration or one major site of inflammation and ulceration extending more than one cm along the length of the colon. Also, the stool consistency (SC) was evaluated visually as the score of 0 (well-formed pellet), 2 (pasty or incompletely-formed stools but not sticking to the anus) and 4 (liquid form sticking to the anus). Moreover, score was set from rectal bleeding (RB) as follows; 0 for no blood, 2 for small bleeding, 4 for gross bleeding. The overall colitis severity, named colitis activity score (CAS), was calculated as follow as the average score of CDS, SC and RB.

The results are shown in Figure 2. For each point, the results were statistically compared with the control group. For CDS, PD and ES-MP did not improve the damage significantly, while normal and Ch-MP/ES showed significantly lower damage score (*p* < 0.01 for normal *vs.* control; *p* < 0.05 for Ch-MP/ES *vs.* control). Ch-MP/ES tended to improve SC more than PD and ES-MP. In RB, PD, ES-MP and Ch-MP/ES exhibited good improvement. As to CAS, only Ch-MP/ES eliminated the severity score significantly (5 mg PD eq/kg: *p* < 0.05 *vs.* control, 10 mg PD eq/kg: *p* < 0.01 *vs.* control).

Furthermore, the efficacy was evaluated based on other indices such as (proximal colon weight)/(body weight), (distal colon weight)/(body weight) and myeloperoxidase activity, which were named as Cp/B, Cd/B, and MPO activity, respectively; that is, Cp, Cd and B represented proximal colon weight, distal colon weight and body weight, respectively [9,10,15,18]. For each point, the results were compared statistically with the control group. Colitis induced a slight elevation in Cp/B, but markedly increased Cd/B and MPO activity. The anti-inflammatory effect found in Cp/B was similar among PD, ES-MP, and Ch-MP/ES. Regarding Cd/B, the suppression of inflammation was obtained significantly by many formulations, and tended to be greater by Ch-MP/ES than by PD and ES-MP. The results of the MPO assay also indicated that Ch-MP/ES tended to decrease MPO activity, whereas PD and ES-MP had negligible effects. These results suggested that the efficacy of Ch-MP/ES should be superior to those of PD and ES-MP. The difference in MPO activity was not significantly, which might be because of the relatively large variance of the data. Overall, the significant differences were not found in Cp/B, Cd/B and MPO activity among formulations due to the fairly large variation of each result; these might need to be investigated in more detail.

Thymus atrophy is often used as a toxic side effect of PD [9,10]. It was observed by the induction of colitis with TNBS (Figure 3D). PD and ES-MP further decreased thymus weight; ES-MP caused the greatest reduction in T/B under the condition of 10 mg/kg × 3 d. On the other hand, Ch-MP/ES did not cause further reductions in thymus weight; significant differences were not obtained due to large variations in the control group. However, it was demonstrated that the toxic side effects tended to be smaller by Ch-MP/ES than by PD and ES-MP.

The results in Figure 2 and Figure 3 demonstrated that Ch-MP/ES should be superior in efficacy and safety to PD and ES-MP.

### 2.3. Gastrointestinal Drug Distribution

The biodisposition features of PD, ES-MP, and Ch-MP/ES were investigated in an attempt to elucidate the mechanisms underlying their efficacies and toxicities in more detail. In order to achieve this, the gastrointestinal distribution profile of PD was examined after the intra-gastric administration of PD, ES-MP, and Ch-MP/ES at 5 mg PD eq/kg to rats with TNBS-induced colitis. The recovery ratio of PD from each segment was examined by the treatment of the sample with known drug concentration and the subsequent assay by HPLC; the recovery ratios of PD were 87%, 87%, 93%, 95%, 93%, and 93% for stomach (S), Upper half small intestine (PI), lower half small intestine (DI), cecum (Ce), proximal colon (upper one third colon, CP), and distal colon (lower two thirds colon, CD), respectively.

**Figure 4:**
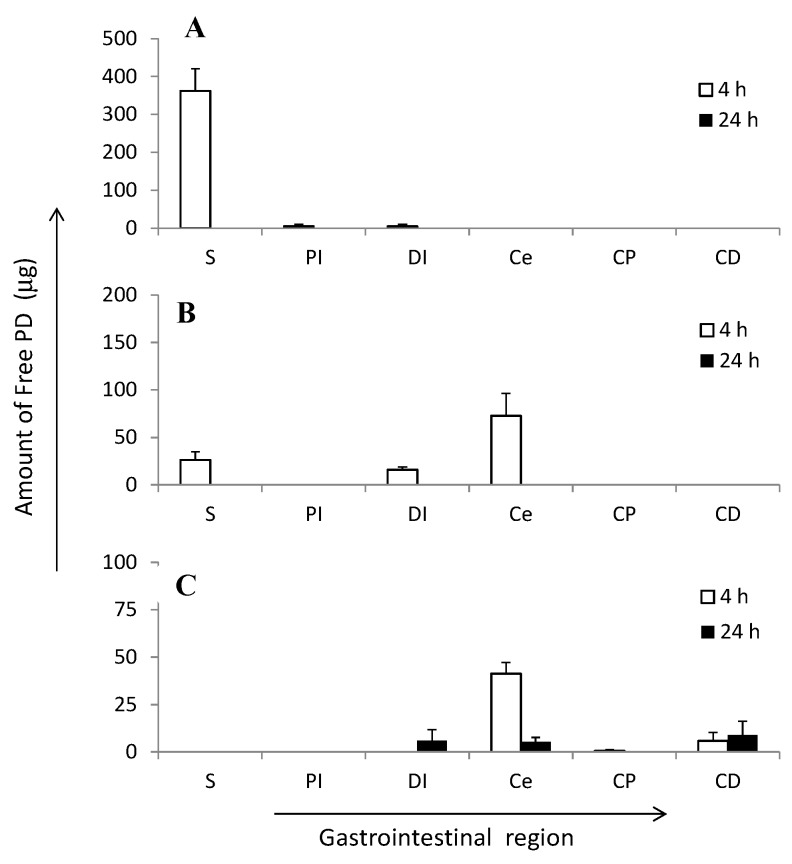
Distribution of free PD in gastrointestinal regions at 4 and 24 h after intra-gastric administration of (**A**) PD; (**B**) ES-MP and (**C**) Ch-MP/ES at 5 mg PD eq/kg in rats with TNBS-induced colitis. The results are described as the mean ± S.E. (*n* = 3).

PD, ES-MP, and Ch-MP/ES showed marked difference in the gastrointestinal distribution (Figure 4). After the ingestion of PD, it was mainly observed in the stomach and to some extent in the small intestine only at 4 h, and was not detected after 24 h. After the intra-gastric administration, ES-MP showed the PD distribution in stomach, lower small intestine and cecum at 4 h. However, PD was not detected in the gastrointestinal tract after 24 h. In the case of ES-MP, although homogenization was only performed for a short period, ES may have been dissolved by that process due to the use of PBS. Therefore, the distributed amount of PD may have included loaded PD in addition to free PD.

The preparation conditions of the tissue samples may have to be examined in more detail in order to distinguish free from loaded PD. After the intra-gastric administration of Ch-MP/ES, PD was mainly detected in the cecum and slightly in the distal colon at 4 h. Furthermore, 24 h after the ingestion, Ch-MP/ES displayed the distribution of PD in the lower small intestine, cecum, and distal colon at a small amount of less than 10 µg. The results indicated that Ch-MP/ES efficiently delivered PD to the lower intestine for a long period. PD was possibly released a little from the conjugate in the preparation of the homogenate sample due to the slow hydrolysis of the ester linkage. Therefore, the distributed amount shown in Figure 4C was considered to be derived from free PD distributed in each part; however, further detailed analyses might be needed.

These gastrointestinal distribution profiles indicated that ES-MP delivered PD to the lower intestine better than PD alone; however, its retention in the lower intestine was not long. The distribution of PD in the colonic part was little detected. On the other hand, Ch-MP/ES specifically delivered PD to the lower intestine. Although PD levels in the lower intestine were not high, the distribution of PD in the cecum and colon last very long (from at least 4 to 24 h). These distribution features of Ch-MP/ES were considered to be advantageous in order to suppress the inflammation associated with colitis and to lower toxic side effects caused by systemic absorption.

### 2.4. Plasma Concentration–Time Profiles

**Figure 5:**
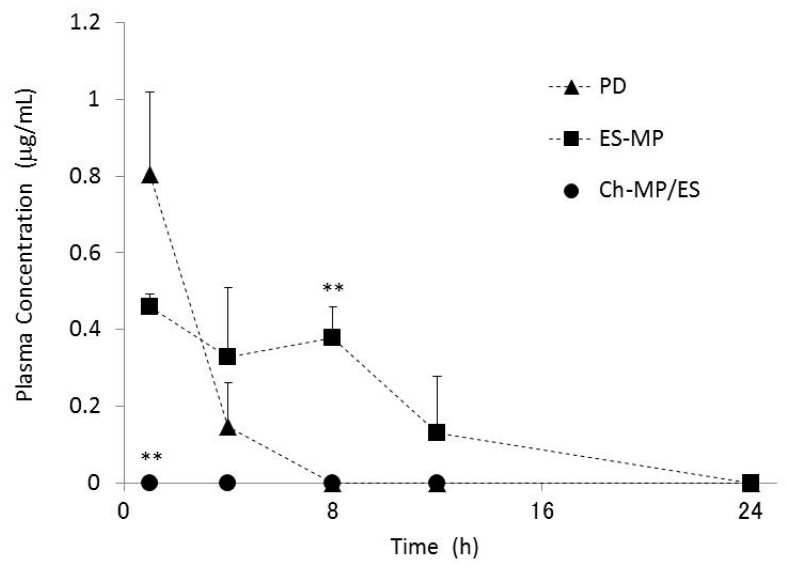
Plasma concentration of free PD after intra-gastric administration of PD, ES-MP and Ch-MP/ES at 5 mg PD eq/kg in rats with TNBS-induced colitis. The results are described as the mean ± S.E. (*n* = 3). ** *p* < 0.01 *vs.* PD.

The diseased model rats with colitis were produced as stated above using TNBS. The plasma concentration of PD was monitored following the intra-gastric administration of PD, ES-MP, and Ch-MP/ES at 5 mg PD eq/kg to the diseased rats (Figure 5). PD exhibited the highest plasma concentration 1 h after its administration, but was eliminated rapidly. PD was not detected from 8 h. Plasma concentrations of PD were lower with ES-MP than with PD in the early stages (1 h); however, ES-MP achieved fairly high concentrations after 4, 8 and 12 h. Following the administration of Ch-MP/ES, PD was hardly detected between 0.5 and 24 h.

These pharmacokinetic profiles indicated that although ES-MP suppressed drug release, PD was gradually and markedly released in the stomach and small intestinal regions, as inferred from previous *in vitro* release profiles [17]. Gastrointestinal distribution (Figure 4) also supported that PD was subjected to gastric and intestinal absorption after the ingestion of ES-MP. The high initial (1 h) level of PD in ES-MP was attributed to its initial small and rapid release in the stomach and the quick transfer of PD and small particles to the intestinal region. Although ES-MP suppressed initial plasma concentrations of PD to some extent, they maintained systemic absorption for a long period of time at a certain level; at 8 h, the plasma concentration of PD was significantly higher with ES-MP than with PD (*p* < 0.01).

On the other hand, Ch-MP/ES suppressed the systemic absorption of PD almost completely. This observation was considered to be due to the very slow release characteristics of Ch-MP/ES [17] and the specific localization to lower intestine, cecum and colon (Figure 4C). Namely, since PD was scarcely observed in stomach and small intestine, PD was presumed to be very little absorbed. On the other hand, the prominent systemic absorption of PD by PD and ES-MP was observed, which would be due to the considerable release and distribution of PD in stomach and small intestine. The great suppression of drug absorption by Ch-MP/ES was consistent with the less thymus atrophy (Figure 3D).

From these *in vivo* results, it was demonstrated that Ch-MP/ES could enhance the efficacy of PD and reduce the toxic side effect of PD, while ES-MP could hardly improve the effects of PD.

## 3. Experimental Section

### 3.1. Materials

Prednisolone (PD) was obtained from Wako Pure Chemical Industries, Ltd. (Osaka, Japan). Chitosan 500 (Ch500; viscosity grade 500 (5 g/L, 20 °C)) was used as chitosan (Ch) in this study. Sodium salt of prednisolone 21-hemisuccinate (SP) sodium salt, named SP-Na, 1-(3-dimethylaminopropyl)-3-ethylcarbodiimide hydrochloride (EDC) and 2,4,6-trinitronenzenesulfonic acid solution (TNBS) were purchased from Sigma-Aldrich (St. Louis, MO, USA). Eudragit S100 (ES) was supplied by Rohm GmbH Chemische Fabrik (Darmstadt, Germany). Sorbitan sesquioleate (SO-15) was purchased from Nikko Chemicals Co., Ltd. (Tokyo, Japan). With respect to other chemicals, compounds of reagent grade were used.

### 3.2. Animals

Male Wistar rats (7 weeks old; weighing 200–210 g) were obtained from Tokyo Laboratory Animals Science Co., Ltd. (Tokyo, Japan), and fed with the breeding diet MF (Oriental Yeast, Co., Ltd., Tokyo, Japan) and water ad libitum at 23 ± 1 °C and relative humidity of 60% ± 5%. They were used in experiments a few days after being purchased. The protocol of the experiment was approved by the Committee on Animal Research of Hoshi University (Tokyo, Japan). Animal experiments were conducted according to the Guiding Principles for the Care and Use of Laboratory Animals in Hoshi University.

### 3.3. Preparation of ES-MP

ES-MP were prepared as described previously [17]. Briefly, ES (180 mg) and PD (20 mg) were dissolved in 5 mL of methanol, and the solution was dripped into liquid paraffin (200 mL) with 1% (*w*/*v*) SO-15, while it was stirred at 1000 rpm. After stirring for 2 h, 1% (*v*/*v*) acetic acid aqueous solution (3 mL) was added, and the stir continued for another 30 min. Then, the mixture was stirred at 1400 rpm under the reduced pressure for several hours, and this operation was continued for further 30 min at 40 °C. After *N*-hexane (200 mL) was added, the resultant mixture was centrifuged at 4500 rpm for 5 min to separate the prodcut. The precipitate was washed with n-hexane, and filtered using a Teflon 0.1 µm pore membrane to collect the product. Finally, the product (ES-MP) was washed with 1% (*v*/*v*) acetic acid aqueous solution, and dried in a desiccator.

### 3.4. Preparation of Ch-MP/ES

Chitosan-succinyl-prednisolone conjugate (Ch-SP) was prepared as described previously [17]. To the queous solution (45 mL) of Ch (120 mg) with the pH adjusted to 3.0 with 1 M HCl aqueous solution, 5 mL of aqueous solution of SP-Na (40 mg) was added, and the pH of the mixture was set at 5.5 using 1 M NaOH aqueous solution. Then, EDC (200 mg) was added, and the solution was stirred at 0 °C for 5 h and at room temperature for another 19 h. Furthermore, after the addition of EDC (200 mg), the reaction was continued for 24 h. After that, acetone was added to precipitate the product, and the precipitate was washed with a mixture of water and acetone (1:4, *v*/*v*). The resultant precipitate was suspended in water and lyophilized into Ch-SP powder.

Next, Ch-SP was processed into the microparticles (Ch-MP) using the emulsification-solvent evaporation method. Namely, after Ch-SP (50 mg) was dissolved in 10 mL of 1% (*w*/*v*) acetic acid aqueous solution, the resultant solution was added gradually to 200 mL of liquid paraffin with 1% (*w*/*v*) SO-15, while the mixture was stirred at 1300 rpm at 40 °C. After the mixture had been stirred for 30 min, it was sonicated under 28 kHz for 10 min and stirred at 1300 rpm for 1 h, and 10 mL acetone was added. Then, the stir was continued at 1300 rpm for 1 h, and the operation was further conducted at 800 rpm under reduced pressure overnight. After the mixture was stirred under reduced pressure at 800 rpm at 40 °C for 30 min, 200 mL of n-hexane had been added, and the product was obtained by centrifugation of the mixture at 4500 rpm for 5 min. The precipitate was washed with n-hexane and dried in a desiccator to obtain Ch-MP powder.

An enteric coating of Ch-MP was conducted using ES. Ch-MP (50 mg) were dispersed in 4 mL methanol solution of ES (100 mg). The suspension was dripped into 200 mL liquid paraffin with 2% (*w*/*v*) SO-15, while the mixture was stirred at 1400 rpm. The mixture was stirred at room temperature under reduced pressure overnight, and the operation was continued at 40 °C for 30 min. Then, n-hexane (200 mL) was added, and the product was obtained as the precipitate. After the precipitate was washed with *N*-hexane, it was dried in a desiccator to yield ES-coated Ch-MP (Ch-MP/ES).

### 3.5. Characterization of Microparticles

The particle sizes, morphologies, and drug contents of ES-MP, Ch-MP, and Ch-MP/ES were investigated. The particles were coated thinly with platinum using a JFC-1600 Auto Fine Coater (JEOL, Tokyo, Japan), and observed by scanning electron microscopy using a JSM-5600LV apparatus (JEOL, Tokyo, Japan). The measurement of Green diameter, being the same as Feret diameter, was performed for the 200 microparticles chosen at random. The particle shapes were also observed from the micrographs.

The drug content of ES-MP was determined as follows. ES-MP (2 mg) were added to 20 mL of a mixed solution of phosphate-buffered saline at pH 7.4 (PBS) and methanol (1:1, *v*/*v*), and the solution was analyzed by HPLC. The HPLC assay was conducted with a LC-6AD pump with a SPD-10AV spectrophotometric detector set at 246 nm. A Supelcosil LC-18-DB (150 mm (length) × 4.6 mm (diameter), filler particle size of 3 μm; Supelco (Bellefonte, PA, USA)) was used as a column, in which the mixture of methanol and PBS (1:1, *v*/*v*) was used as a mobile phase. The drug content of ES-MP was calculated from the concentration of PD. As to Ch-MP and Ch-MP/ES, their drug contents were determined as follows. Microparticles (2 mg) were placed in 10 mL of 0.1 M NaOH aqueous solution, and the mixture was incubated at 45 °C for 10 min. Immediately after that, the mixture was centrifuged to separate PD completely released from the particles. The concentration of the released PD was determined from the UV absorbance of the supernatant. The drug contents were calculated from the concentrations of released PD.

### 3.6. In Vivo Examination of Efficacy and Toxicity

**Figure 6:**
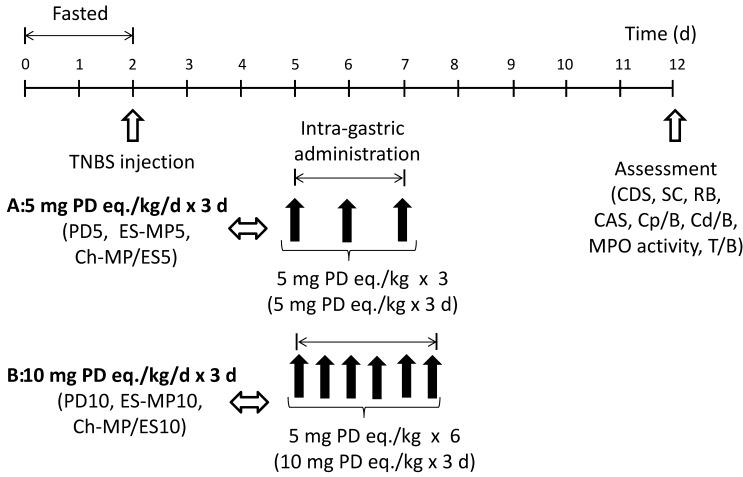
Schedules of studies of efficacy and toxic side effect in rats with TNBS-induced colitis.

Animal experiments were performed as shown in Figure 6 in order to examine the efficacies and toxicities of PD, ES-MP and Ch-MP/ES. The animal models with colitis were produced as follows. To the rats having been fasted for 48 h, TNBS (20 mg) dissolved in 0.25 mL of 50% (*v*/*v*) ethanol was injected into each rat 7 cm from the anus with a catheter [9,10,18,19]. Then, the rats were housed for 3 days without any treatment in order to allow a full inflammation to develop. The rats that developed colitis were divided to seven groups (*n* = 3 in each group) as follows: Control (non-treated), PD5 (5 mg PD/kg × 3 times (every 24 h)), PD10 (5 mg PD/kg × 6 times (every 12 h)), ES-MP5 (5 mg PD eq/kg × 3 times (every 24 h)), ES-MP10 (5 mg PD eq/kg ×6 times (every 12 h)), Ch-MP/ES5 (5 mg PD eq/kg × 3 times (every 24 h)), and Ch-MP/ES10 (5 mg PD eq/kg × 6 times (every 12 h)). In these groups, the substance corresponding to 5 mg PD eq/kg was suspended in 1.5 mL of saline and administered via gastric intubation according to the dosing schedules shown in Figure 6. The healthy rats, not treated with TNBS, were added in the *in vivo* tests as the normal group, named Normal. No substance was ingested in Normal. The *in vivo* efficacy and toxicity of each formulation were evaluated after TNBS was injected (Figure 6).

First, the efficacy was investigated by visually observing the effect of formulations on the severity of colitis. The damage and inflammation of the distal colon were observed visually, and the degree of severity was scored by referring to the criteria of Lamprecht *et al.* [13] and Tozaki *et al.* [18]. A colonic damage score was defined as follows; 0 for no damage, 1 for localized hyperemia but no ulcer, 2 for linear ulcers with no significant inflammation. 3 for linear ulcer with inflammation at one site, 4 for two or more sites of ulceration and/or inflammation, 5 for two or more major site of inflammation and ulceration or one major site of inflammation and ulceration extending more than one cm along the length of the colon. In addition, the colitis severity was examined from stool consistency (SC) and rectal bleeding (RB). The stool consistency (SC) was evaluated as follows; 0 for well-formed pellet), 2 for pasty and incompletely-formed stools not sticking to the anus, 4 for liquid stools sticking to the anus. The rectal bleeding (RB) was dealt with as follows; 0 for no blood, 2 for finding blood, 4 for gross bleeding. Furthermore, the overall colitis severity, named colitis activity score (CAS), was calculated as follow as the average score of CDS, SC and RB.

Next, the efficacies were examined using other indices of colonic inflammation degree, and toxic side effect was investigated using thymus atrophy, often used as one of the PD side effects. Body weight (B) was measured after the sacrifice of animals with excessive ether anesthesia on that day (10 days after TNBS injection), and then the colon and thymus were taken. The colon was cut into 2 parts, that is, the proximal colon (4 cm colonic segment from the end of the cecum) and distal colon (8 cm colonic segment next to the proximal colon) [10,13]. These parts were washed with saline, and the colonic contents were removed. The resultant proximal colon weight (Cp) and distal colon weight (Cd) were measured, and the ratios, Cp/B and Cd/B, were calculated. Furthermore, MPO activity in the distal colon, often used as an index of inflammation [10,13], was investigated using an MPO assay kit (Cytostore, AB, Canada) as follows. After the distal colon was washed with saline and its content was removed, a specimen (200 mg) of the inflammation site was taken and homogenized in 0.5% (*w*/*v*) hexadecyltrimethylammonium bromide (HTAB) aqueous solution on ice, and diluted to 4 mL with HTAB aqueous solution. After the suspension was sonicated for 10 s, the operation of freeze-thaw was repeated three times. Then, the resultant suspension underwent vortex mixing, and centrifuged at 10,000 rpm for 3 min. The supernatant (200 µL) was mixed with 2 mL of the kit development reagent containing o-dianisidine dihydrochloride at 0.167 mg/mL and hydrogen peroxide at 0.005 mg/mL at 25 °C. Absorbance was then measured at 450 nm. After the mixture had been maintained at 25 °C for 60 s, absorbance was again measured at 450 nm. The difference in absorbance (450 nm) between 0 s and 60 s was used as MPO activity. Furthermore, the thymus was removed and washed with saline. From tts weight (T) and body weight (B), the T/B ratio was calculated to evaluate the toxic side effects.

### 3.7. Investigation of Gastrointestinal Drug Distribution

This experiment was performed using diseased rats with TNBS-induced colitis, which was produced as described above. Namely, three days after the colonic injection of TNBS, the rats were fasted for 48 h. PD, ES-MP, and Ch-MP/ES were ingested intra-gastrically at 5 mg PD eq/kg, in which the drug sample suspension was administered at a total volume of 1.5 mL per rat. After 4 and 24 h, the rats were sacrificed by excessive anesthesia with ethyl ether gas. At each time, the stomach (S), upper half of the small intestine (PI), lower half of the small intestine (DI), cecum (Ce), upper one third of the colon (CP), and lower two-thirds of colon (CD) were taken. Each tissue was homogenized with its content under the condition of ice cooling, in which a glass homogenizer was used with a Teflon pestle. The homogenate of CP was diluted to 4 mL with PBS. As to other tissues, the homogenate was diluted to 8 mL with PBS. The extraction of PD from each diluted homogenate was conducted as follows. Saturated NaCl aqueous solution (100 µL), 5% (*w*/*v*) phosphoric acid (100 µL) and 4 mL of the mixture of *t*-butylmethyl ether and pentane (3:2, *v*/*v*) were added to 100 µL of the final tissue homogenate, and the resultant mixture was vigorously shaken. The resultant organic phase (2 mL) was withdrawn and dried under nitrogen gas at room temperature. The obtained residue was dissolved in the mobile phase in HPLC assay, and the PD concentration was determined by HPLC, in which the experimental condition was the same as that in the drug content study stated above, except that 22% (*v*/*v*) 2-propanol aqueous solution with 0.1% (*v*/*v*) trifluoroacetic acid was used as a mobile phase. The recovery study was performed by adding a known amount of PD to a drug-free tissue homogenate in the range around the observed concentration. The recovery ratio was determined as the ratio of the observed amount to the calculated amount, and the data was corrected using that ratio.

### 3.8. Examination of Plasma Concentrations–Time Profiles

Drug administration was performed in the same way as stated above in the gastrointestinal distribution study. Namely, three days after the colonic instillation of TNBS, the rats were fasted for 48 h. Then, PD, ES-MP, and Ch-MP/ES were administered using gastric intubation at 5 mg PD eq/kg with a 1.5 mL saline suspension per rat. Immediately before and 1, 4, 8, 12, and 24 h after this administration, blood (0.5 mL) was taken from the jugular vein under the light anesthesia by ether inhalation. After the plasma was obtained by centrifugation of the blood, 100 µL of plasma was extracted in the same manner as described for the above final tissue homogenate. The final residue, obtained from the organic phase, was dissolved in the mobile phase of HPLC, and determined for the PD amount by HPLC, the condition of which is the same as described in the investigation of gastrointestinal drug distribution. The recovery study was conducted by adding a known amount of PD to drug-free plasma in the range around the observed concentration. The recovery ratio was determined by comparison of the observed amount with the calculated amount.

### 3.9. Statistical Analysis

Data were analyzed statistically using ANOVA and subsequent post hoc test with the Dunnett’s method. A significant difference was judged as *p* < 0.05.

## 4. Conclusions

Efficacy, toxic side effects, and pharmacokinetic characteristics of the small microparticle systems, ES-MP and Ch-MP/ES, were compared *in vivo*. Although ES-MP exhibited pH-dependent release based on ES features, their *in vivo* efficacy and toxic side effects were not different from those of PD. Furthermore, delivery to the lower intestine was achieved to a certain extent by ES-MP, but the fairly high plasma concentration of PD was found from 0 to 12 h. On the other hand, Ch-MP/ES promoted *in vivo* efficacy and ameliorated the severity of toxic side effects, and these results were attributed to marked changes in gastrointestinal distribution and the complete suppression of the plasma concentration. Although ES-MP changed the release profile *in vitro* from that of PD, that modification was not enough to improve *in vivo* function. Only Ch-MP/ES, which markedly changed *in vitro* and *in vivo* features, were found to improve the *in vivo* function of PD. Further investigations might be needed for more exact evaluations.
